# Evolution of Developmental GATA Factors in Nematodes

**DOI:** 10.3390/jdb8040027

**Published:** 2020-11-16

**Authors:** Ethan Eurmsirilerd, Morris F. Maduro

**Affiliations:** 1 Undergraduate Program in Biology, Department of Molecular, Cell, and Systems Biology, University of California, Riverside, Riverside, CA 92521, USA; eeurm001@ucr.edu; 2 Department of Molecular, Cell, and Systems Biology, University of California, Riverside, Riverside, CA 92521, USA

**Keywords:** GATA factors, transcription factors, nematodes

## Abstract

GATA transcription factors are found in animals, plants, and fungi. In animals, they have important developmental roles in controlling specification of cell identities and executing tissue-specific differentiation. The Phylum Nematoda is a diverse group of vermiform animals that inhabit ecological niches all over the world. Both free-living and parasitic species are known, including those that cause human infectious disease. To date, GATA factors in nematodes have been studied almost exclusively in the model system *C. elegans* and its close relatives. In this study, we use newly available sequences to identify GATA factors across the nematode phylum. We find that most species have fewer than six GATA factors, but some species have 10 or more. Comparisons of gene and protein structure suggest that there were at most two GATA factors at the base of the phylum, which expanded by duplication and modification to result in a core set of four factors. The high degree of structural similarity with the corresponding orthologues in *C. elegans* suggests that the nematode GATA factors share similar functions in development.

## 1. Introduction

**Figure 1:**
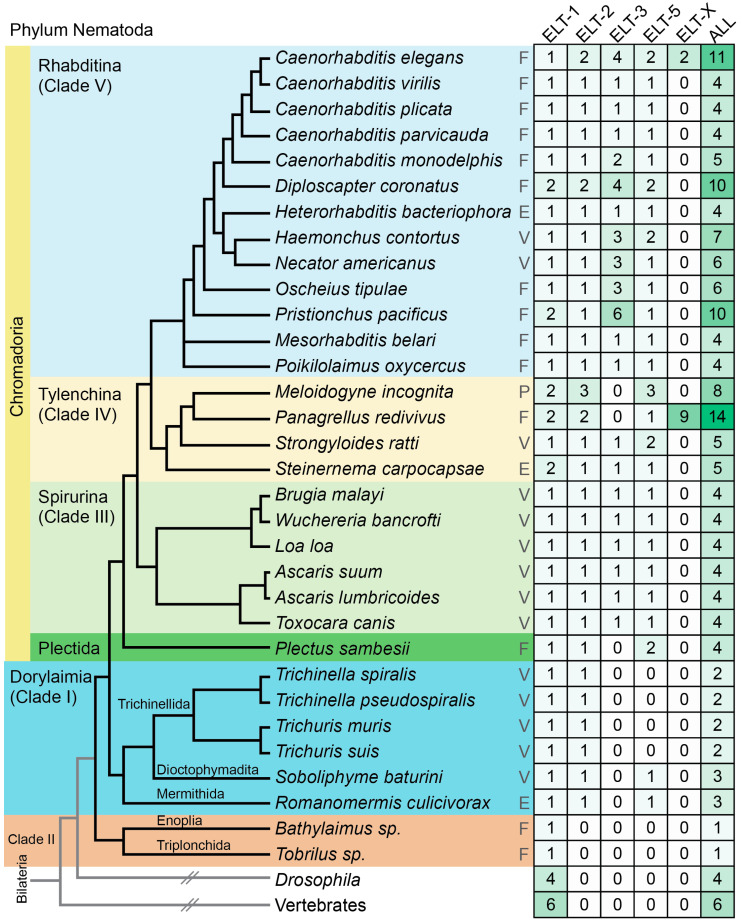
Phylogeny of selected species from the Nematode phylum and numbers of GATA factors. Phylogenetic relationships were obtained from prior work [2,47,56,57,58]. The life histories are abbreviated as follows: F, free-living; E, entomopathogenic; V, vertebrate parasite; P, plant parasite. The number of GATA factors of each type, and the total number of factors, are shown in the table on the right side. *nd*, not determined. Higher numbers have darker green shading behind them.

GATA factors are an ancient family of eukaryotic transcription factors that mediate processes of development, differentiation, and gene expression in multiple cells and tissue types. The Nematode Phylum comprises a rich and varied suite of species occupying varying ecological niches, including free-living, entomopathogenic, and animal and plant parasitic [1]. Ribosomal RNA analysis has subdivided the phylum as shown in Figure 1, and we will use the simpler five-clade taxonomy [2]. The major groups are the Chromadorea, which includes Clade III (Spirurina), Clade IV (Tylenchina) and Clade V (Rhabditina, which includes *C. elegans*); Dorylaimia, or Clade I; and Enoplia and Triplonchida, representing Clade II. An additional group, the Plectids, is a sister group to Clades III, IV, and V within the Chromadorea [3]. Nematodes are considered as a sister group to the Arthropods, and grouped together as a clade of molting animals, or Ecdysozoa [4].

Juvenile and adult nematodes are superficially similar in morphology across the phylum, although embryonic development has been found to vary [5]. The best-studied model, *C. elegans*, exhibits a largely invariant cell lineage in which cell fates are apportioned early to a set of founder cells [6]. Other species have surprising differences from *C. elegans*, including different lineage patterns and changes in the division axes of cells, and differences in cell movements in gastrulation [7,8,9,10]. These differences in early development suggest that early developmental regulators might vary considerably across the phylum; however, those that act in later stages may be conserved. The identification of classes of transcription factors may reveal, therefore, by sequence similarity, patterns of conserved regulators of development.

GATA factors consist of Cys_2_-Cys_2_ (C4) zinc finger DNA-binding domains that recognize sequences whose core is HGATAR [11]. In fungi, GATA factors regulate aspects of nitrogen metabolism [12,13]. In plants, GATA factors regulate processes such as photosynthesis, growth, and metabolism [14,15,16]. In animals, GATA factors act primarily during development [17,18]. In *Drosophila* they are important in endoderm development, hematopoiesis, and neural development [19,20,21]. In humans, GATAs are important for cell specification and tissue morphogenesis across multiple tissue types and mutations in these contribute to genetic diseases and birth defects [18,22]. 

GATA factors in plants and fungi generally have one DNA-binding domain (DBD) consisting of a C4 zinc finger and associated basic domain, while those in vertebrates and arthropods generally have two DBDs in tandem [11,23,24,25,26]. In vertebrates, the GATA factors are often divided into the ‘GATA123’ and ‘GATA456’ classes, with the former functioning in mesoderm and ectoderm tissues, and the latter in endoderm and mesoderm [26,27]. In these two-finger vertebrate GATA factors, the carboxyl DBD mediates sequence-specific binding to a GATA site, while the amino zinc finger and basic domain provide a reinforcing DNA-interacting role [28,29]. In the longest isoform of the *Drosophila* GATA factor *Serpent*, the amino zinc finger stabilizes interaction with palindromic GATA target sites and allows protein-protein interactions with the protein U-shaped [30]. Hence, in both vertebrate and *Drosophila* GATA factors, it is the carboxyl DBD that mediates sequence recognition of the target HGATAR site.

The best studied GATA factors in nematodes are those of *C. elegans*. Among 11 GATA factors, only the ELT-1 (Erythroid-Like Transcription) factor has two DNA-binding domains, while the remainder have only one. The ELT-2 protein has an apparent ‘degenerate’ upstream C4-type zinc finger. As in humans and arthropods, the *C. elegans* GATA factors act early in cell specification and later in tissue differentiation [31,32,33,34,35,36]. Four GATA factors, MED-1 (Mesoderm and Endoderm Determining), MED-2, END-1 (ENdoderm Determining), and END-3, are transiently expressed in the early embryo where they act to specify the MS (mesoderm) and E (endoderm) blastomeres [37]. MED-1,2 specify both MS and E, while END-1,3 are targets of MED-1,2 and specify only E. Unlike the MED and END factors that are activated in the early embryo only transiently, the other *C. elegans* GATA factors are activated later in embryogenesis and remain expressed for the lifetime of the animal [32,33,34,35,36,38]. The GATA factors ELT-2, ELT-4 and ELT-7, are also expressed in the early endoderm. Only ELT-2 has an essential function [32]; ELT-4 is a nonfunctional partial duplication of ELT-2, while ELT-7 reinforces ELT-2 but is otherwise dispensable for normal development [38,39]. Collectively, all of MED-1/2, END-1/3, ELT-4 and ELT-7 are derived among nematodes, as they are found only in the Elegans Supergroup consisting of *C. elegans* and its close relatives [40]. The remaining GATA factors in *C. elegans* are ELT-1 and ELT-3, which have overlapping function in differentiation of the epidermis, while EGL-18 (originally called ELT-5) and its paralog ELT-6 function in late specification of a subset of epidermal cells [31,33,34]. 

A prior study examined nematode GATA factors in the context of GATA factor evolution among the Protostomes; however, that work considered factors only in *C. elegans* and its close relative *C. briggsae* because they were the only species whose sequences were available [25]. To understand the evolution of all GATA factors among nematodes in general, we compared predicted GATA factors in their gene and protein structures for 32 species across the phylum for which there is now sequence information. Our results suggest that, in general, nematode genomes encode fewer than 6 GATA factors, with expansion occurring only rarely as in *C. elegans*. The high degree of conservation in the DNA-binding domains suggests these factors have maintained similar developmental roles throughout evolution.

## 2. Materials and Methods

### 2.1. Identification of GATA Factors in Nematode Species

BLAST searches were performed using default settings [41]. To identify putative GATA factors in a species, BLASTP searches were performed using the NCBI BLAST server [42]), Wormbase [43], the Caenorhabditis Genomes Project [44], and Command Line BLAST+ 2.7.1 and 2.10.1 executables running in Windows PowerShell (PC) or Terminal (Apple Macintosh), from https://ftp.ncbi.nlm.nih.gov/blast/executables/blast+/LATEST/. We generally searched with the carboxyl zinc finger and basic domain of an ELT-1 orthologue from the same or neighboring clade, though searching with most GATA type zinc finger and basic domains retrieved the same set of genes. For the expanded set of single-finger GATA factors in *P. redivivus*, we provisionally assigned these to an ‘ELT-X’ class. Gene models and protein sequences were examined in ApE v2.0.61 (https://jorgensen.biology.utah.edu/wayned/ape/) or Vector NTI 6 (Informax). Intron positions were deduced from CDS coordinates, or, in the case of cGATA1, by TBLASTN search of the protein to genome sequences of *G. gallus.* In addition to BLASTP, we searched genome sequences by TBLASTN to confirm that all putative GATA-type zinc fingers had been identified. In some cases, we identified putative GATA factors that were completely missed by gene predictions, such as two missed GATA factors in *P. redivivus*. We also found that altered splicing patterns could often restore an apparently incomplete predicted protein. For example, in *N. americanus*, the carboxyl end of the zinc finger of a putative ELT-2 ortholog was missing the fourth cysteine and lacked conservation within what should have been the adjacent putative basic domain. A change in the predicted splicing in this region resulted in a predicted protein whose carboxyl end contained the fourth cysteine and conserved basic domain and was conserved among ELT-2 orthologs. Partial zinc fingers for which no coding potential could restore a complete structure were treated as pseudogenes and left out of the analysis. Where multiple overlapping predicted transcripts (and hence protein sequences) existed for a single putative gene, we arbitrarily chose the lowest-numbered one that contained intact one or more intact DNA-binding domains. Although some additional GATA factors may remain unidentified, or predictions of protein sequence outside of the well-conserved DNA-binding domains are incorrect, such inaccuracies would not likely affect the major conclusions of this study.

### 2.2. Assignment of Putative GATA Factors to Classes

Based on sequence homology and the number and type of zinc fingers, we were able to classify GATA factors in one of five classes: ELT-1-like, which contained two adjacent DNA-binding domains that strongly resembled those found in *C. elegans* ELT-1, and which resembled GATA factors in arthropods and vertebrates; ELT-2-like, which contained an upstream CX_2_C-X_9–20_CX_2_C zinc finger-like domain of limited conservation of variable structure and a GATA-type DNA-binding domain conserved with *C. elegans* ELT-2; single DNA-binding domain GATAs that were more closely related to *C. elegans* ELT-3 or ELT-5; and for *P. redivivus*, which showed a diverse set of extra GATA factors that were not easily classified, we placed some factors in an ‘ELT-X’ category. Classification in some cases was arbitrary because of similar sequence similarity to multiple classes of factor. We expect that some factors may be mis-classified because of incorrect or incomplete sequence predictions. For example, the *S. baturini* putative ELT-2 orthologue shares some additional conservation with both ELT-1 and ELT-2 in the amino C4 domain, so it shares structural features with both ELT-1 and ELT-2. Finally, in *C. elegans*, we classified ELT-6 as an ‘ELT-5’ factor because ELT-5 and ELT-6 are paralogous. For the RAxML tree analysis, we omitted the derived *C. elegans* MED-1, MED-2, END-1, and END-3 factors which are found only in close relatives of this species [40].

### 2.3. Reference Genome Sequences

Genome sequence scaffolds, gene models, and protein predictions were retrieved from WormBase ParaSite [45] and the Caenorhabditis Genomes Project [44]. Reference versions were as follows: *C. elegans*, WS277; *C. virilis*, JU1968_v1; *C. plicata*, SB355_v1; *C. parvicauda* NIC534_v1; *C. monodelphis*, JU1667_v2; *D. coronatus,* PRJDB3143.WBPS14; *H. bacteriophora,* M31e_PRJNA13977; *O. tipulae*, CEW1_nOt2; *P. pacificus*, PRJNA12644.WBPS14; *M. belari*, JU2817_v2; *P. oxycercus*, v1; *M. incognita,* PRJEB8714.WBPS14; *P. redivivus*, PRJNA186477.WBPS14; *S. ratti*, PRJEB125.WBPS14; *S. carpocapsae*, PRJNA202318.WBPS14; *B. malayi*, PRJNA10729.WBPS14; *W. bancrofti*, PRJEB536.WBPS14 and PRJNA275548.WBPS14; *Loa loa*, PRJNA37757.WBPS14 and PRJNA246086.WBPS14; *A. suum*, PRJNA80881.WBPS14 and PRJNA62057.WBPS14; *A. lumbricoides*, PRJEB4950.WBPS14; *T. canis*, PRJEB533.WBPS14 and PRJNA248777.WBPS14; *P. sambesii*, PRJNA390260.WBPS14; *T. spiralis*, PRJNA12603.WBPS14 and PRJNA257433.WBPS14; *T. pseudospiralis*, ISS176_PRJNA257433.WBPS14; *T. muris*, PRJEB126.WBPS14; *T. suis*, PRJNA179528.WBPS14; *S. baturini*, PRJEB516.WBPS14; *R. culcivorax*, PRJEB1358.WBPS14. For retrieval of 5′ and 3′ flanking sequences, we used the WBPS15 versions of the sequence scaffolds and gff3 files for these species: *H. contortus*, *N. americanus*, *S. ratti*, *S. carpocapsae*, and *T. canis*. References for most of these sequences appear on WormBase Parasite. References for recent sequences retrieved from the Caenorhabditis Genomes Project include those of *C. monodelphis* [46], *H. bacteriophora* [47], *M. belari* [48], and *P. oxycercus* [49].

### 2.4. Construction of Alignments and Relatedness Trees

Protein alignments were initially performed using MUSCLE within MEGA-X [50]. Trees were constructed using RAxML-NG v0.9.0 at https://raxml-ng.vital-it.ch [51] or the CIPRES science gateway, https://www.phylo.org/ [52]. Trees were converted to SVG format using IcyTree, https://icytree.org [53].

### 2.5. Identification of Putative Regulatory Sequence Motifs

Fasta files of up to 1000 bp of 5′ and 3′ flanking sequence were generated from gff3 and scaffold sequence data and processed using Multiple Em for Motif Elicitation (MEME)5.2.0 at https://meme-suite.org [54]. Retrieved sequences are in Supplemental File S1. For 5′ flanking sequences (putative promoters), we used a 2-order background model with any number of repeats and a motif size of 6-15 bp. For 3′ sequences (putative 3′UTRs), we used the same settings except for requesting a motif size of 6-25 bp. Alternate settings produced similar results. We used one promoter per GATA factor class. Where there were multiple paralogs, we arbitrarily chose one of them as a representative. We omitted the Clade II species for this analysis because these were not genomic DNA sequences. A major caveat of these putative flanking regions is that, outside of the sequence encoding the conserved DNA-binding domains, the predicted 5′ and 3′ ends of entire open reading frames are likely to be unreliable for at least some of the genes.

### 2.6. Additional Computational Resources

Data were assembled in Microsoft Excel and figures generated in Adobe Illustrator. Position-weighted matrices for DNA-binding sites were obtained from the CISBP website [55]. Identification of zinc fingers and basic domains, conversion of data formats, retrieval of General Feature Format (GFF) and nucleotide sequence data, and generation of diagrams of protein structure, were partially automated by using programs written in Python 3.7.1.

## 3. Results

### 3.1. GATA Factors Are Found across the Nematode Phylum

**Figure 2:**
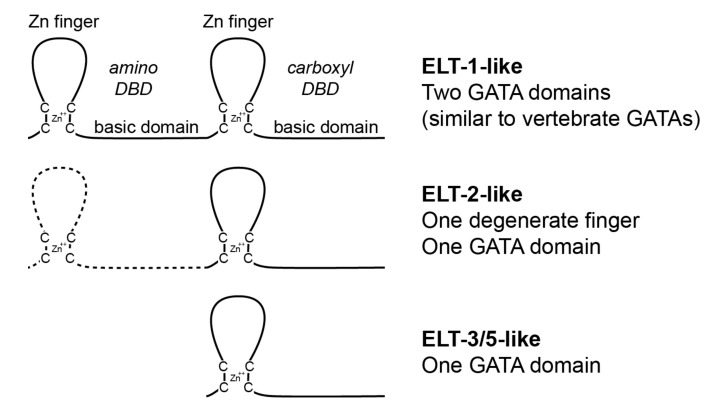
Three major GATA factor classes are found in nematodes. The canonical DNA-binding domain (DBD) of GATA factors consists of a C4-type zinc finger and associated basic domain. The Erythroid-Like Transcription (ELT)-1 type of factor has two DBDs in tandem; the ELT-2 type has a divergent (and degenerate) C4-type structure, and one GATA DBD; and the ELT-3/5 type has a single DBD. Of the three types, only the ELT-1 type is conserved outside of nematodes.

We identified GATA factors from available genome sequences of nematode species. The factors sorted into five categories based on the number of DNA-binding domains and similarity to GATA factors in *C. elegans* (see Materials and Methodsand Supplemental File S1). A tally of the number of factors in each species is shown in Figure 1 and the general structures of the factors are shown in Figure 2. We found that, except for Clade II, all species have at least two GATA factors, an ELT-1 and an ELT-2. Within the Dorylaimia (Clade I), orthologues of ELT-1/2 are the only GATA factors among the Trichinellida, while *S. baturini* and *R. culicivorax* each have one of ELT-1, ELT-2, and ELT-5. Species of the Spirurina (Clade III) have four GATA factors, with a single representative each of ELT-1, ELT-2, ELT-3, and ELT-5. The Plectid *P. sambesii* is similar but lacks an ELT-3 and has two putative ELT-5 orthologues.

Within the Chromadoria, species in the Tylenchina (Clade IV) and Rhabditina (Clade V) encode more GATA factors on average. Although about 1/3 of the species have four GATAs like the Spirurina (Clade III), the rest encode at least one more. In some cases, additional GATAs are apparent paralogous duplicates of ELT-1 and/or ELT-2, but most of the additional factors are of the single-finger type. Several species have at least 10 GATA factors: In addition to *C. elegans*, which encodes 11 GATA factors, there are *Diploscapter* with 10 factors, *Pristionchus* with 10 (all three species in Clade V), and *Panagrellus redivivus* with an unexpected number of 14 factors. Species with 10 or more GATA factors happen to be free-living; however, there may be too few sampled parasitic nematodes among Clades IV and V to reach a firm conclusion.

Within the Elegans Supergroup of closely related species, we previously observed synteny of recently evolved paralogous genes, consistent with recent linked duplication [40]. This includes the genes encoding the paralogous END-1 and END-3 factors in *C. elegans* and other species, as well as many examples of linked MED duplicates. Apart from *C. elegans,* among the 19 cases where we identified at least two paralogous factors in this work, we found 6 examples of gene pairs separated by 2.6–35 kbp, and an additional pair of paralogues separated by 893 kbp. Of these 7 pairs, 5 are transcribed in the same direction, while 2 pairs are in convergent and inverted orientation (shown diagrammatically in Figure S1). Hence, at least ~40% of the GATA factor paralogues are nearby in their respective genomes. Additional synteny cannot be ruled out as most of the genome sequences are not complete to the level of intact chromosomes; hence, linked paralogues could be on different scaffolds that have not been joined.

Last, we identified a putative ELT-1-like factor in *Tobrilus sp.* and part of an ELT-1-like factor in *Bathylaimus* sp. from transcriptional data [57]. The basic domains of the amino DBD in the *Tobrilus* sp. ELT-1, and the DBD in the *Bathylaimus* DBD, are divergent from those of the other nematode GATAs (see next section). Furthermore, we cannot rule out the occurrence of other GATA factors in these species, as low-expressed GATA factors, or those expressed outside of life stages used for mRNA purification, could have been missed. Consistent with this, these two GATA factors were the only ones obtained out of five species transcriptomic datasets available for Clade II [57].

### 3.2. GATA Factor Sequences across the Phylum Tend to Be Related by Clade

**Figure 3:**
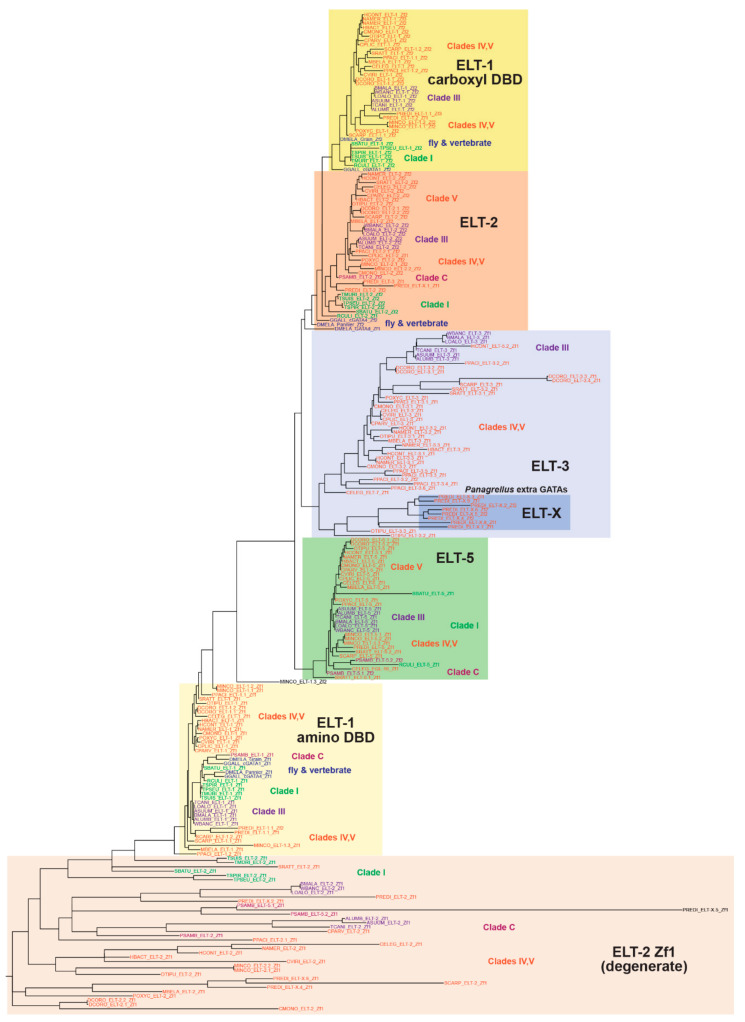
Phylogenetic tree of the DNA-binding domains of GATA factors from nematodes, and from representative GATAs of vertebrates (chicken GATA1 and GATA4) and *Drosophila* (*Pannier*, *Grain*, and *GATAd*). For this analysis we omitted the derived *C. elegans* factors ELT-4, MED-1/2, and END-1/3. The tree was constructed by aligning the DBDs using MUSCLE in MEGA-X and using these to generate a tree using RAxML-NG [50,51]. Broad classes of factors have been indicated with colored boxes and classified by the zinc finger (Zf) associated with the DNA-binding domains. The non-nematode GATAs, which align most strongly with the ELT-1 orthologues, have been indicated. Colored text is used to indicate factors by clade.

To deduce possible evolutionary origins of the nematode GATA factors, we constructed a maximum-likelihood tree using RAxML-NG [51]. Because of high divergence outside of the DNA-binding domains, we used the C4 zinc fingers and downstream 30 amino acids to capture the associated basic domain. To look for possible conservation in the degenerate amino zinc finger in the ELT-2 orthologues, we included these and the adjacent amino acids, as well. To determine orthology with GATA factors outside of the phylum, we included representative vertebrate GATA factors, chicken GATA1 and GATA4, and three from *Drosophila*, the two-DBD *Pannier* and *Grain* and the single-DBD *GATAd*. The resulting tree is shown in Figure 3. In this tree, we omitted the highly divergent sequences from *Tobrilus* sp. and *Bathylaimus* sp.; when these are included, the overall structure of the tree becomes highly fragmented. An example of such a tree is shown in Supplementary Figure S2.

Overall, the DNA-binding domains, and the degenerate zinc finger of the ELT-2 orthologues, cluster together as expected. The upstream zinc fingers found in most ELT-2 orthologues were loosely grouped together at the base of the tree and exhibited exceptionally long branch lengths compared to the other factors, demonstrating their limited conservation. The degenerate ELT-2 zinc finger of closely related species, such as the two *Ascaris* species and *Toxocara canis*, tended to cluster together.

The two DNA-binding domains of ELT-1 each formed their own clusters, with only one exception, with an isolated second DBD of *M. incognita* ELT-1.3. Within both, the DBDs of more closely related nematodes tended to cluster together, especially those of Clades I and III. The amino DBD of the *Drosophila* and chicken GATA factors were found within the ELT-1 amino DBD cluster, consistent with their being homologous. Within the cluster containing the carboxyl DBDs of the ELT-1 orthologues, the carboxyl DBDs of *Drosophila Grain* and chicken cGATA1 were also found. The ELT-2 DBD cluster was next to the ELT-1 second DBD cluster, consistent with its highly similar sequence, and it was also close to the corresponding fly and vertebrate GATA DBDs.

The remaining two types of GATA factor form related clusters as expected. The ELT-5 cluster is very well conserved overall, except for the divergent Clade I *R. culicivorax* and *S. baturini* ELT-5 DBDs. In contrast, the ELT-3 DBDs are a much more divergent group overall. The longer branch lengths are consistent with relaxed constraints on conservation of the amino acid sequences compared with ELT-5. Within the ELT-3 group are most of the extra GATA factors that were found in *P. redivivus*, consistent with these having evolved by duplication of an ELT-3 progenitor, with further duplication and sequence divergence over time.

The results from the RAxML tree indicate that, among the DNA-binding domains, the vast majority are well-conserved, with more divergence evident among the ELT-3 orthologues as a whole.

### 3.3. Comparison of Nematode GATA Factor Structures

**Figure 4:**
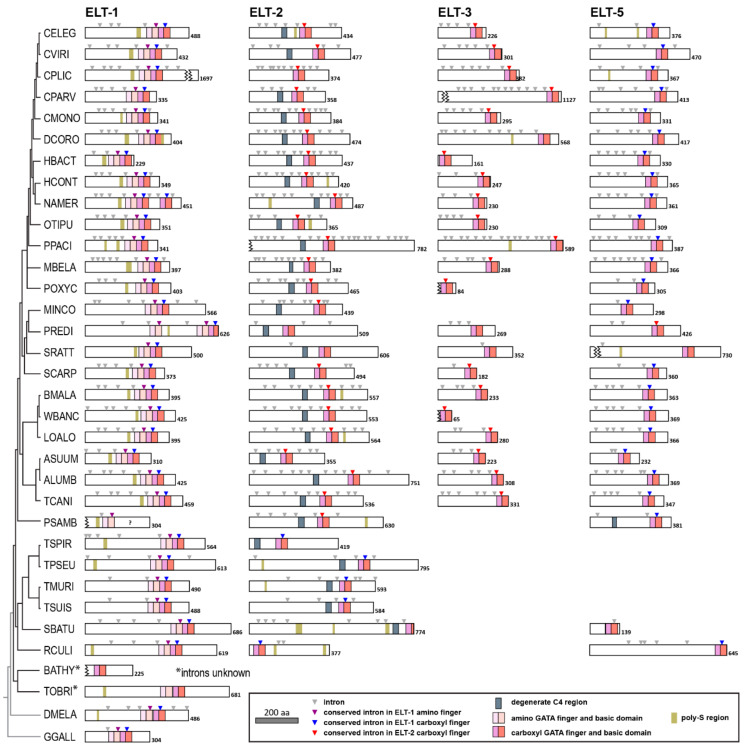
Structures of predicted GATA factors in nematodes. The relationship diagram and species order top to bottom are the same as in Figure 1. Where we found more than one protein in a particular class, we arbitrarily chose one. DNA-binding domains (zinc finger and basic domains), the degenerate C4 domain, and poly-serine domains are shown by colored shading as indicated in the legend. Intron positions are shown as inverted triangles on top of the proteins. A subset of these has been color-coded to indicate conservation by relative position in the coding region. The total number of amino acids is shown for each protein. Except for a few proteins that have been truncated for space, all are drawn to scale. In these cases, and for proteins where the sequence is only partial, we have indicated missing parts of the protein with a jagged edge.

**Figure 5:**
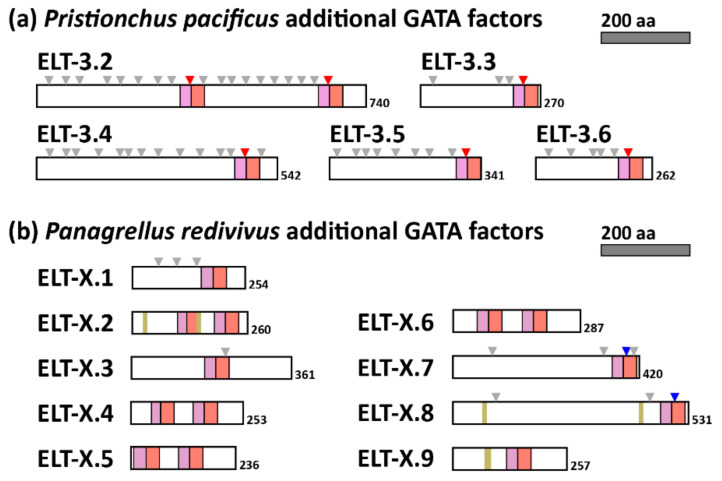
Diagrams of extra GATA factors encoded in the *Pristionchus pacificus* (**a**) and *Panagrellus redivivus* (**b**) genomes. Symbols and colors are as in Figure 4.

To obtain further insight into the evolutionary dynamics of the nematode GATA factors, we compared their structures, as shown in Figure 4 and Figure 5. The results reveal conserved features within each class of factor. In the case of ELT-1, the majority (29/32) have two adjacent zinc fingers in tandem, like *C. elegans* ELT-1 and the *Drosophila* and chicken GATA factors. In *P. redivivus*, a duplication of the upstream DBD has occurred, and in *Necator americanus*, the carboxyl DBD that has been duplicated, hence these ELT-1 orthologues each have three apparent zinc fingers. In the third case, the Plectid *P. sambesii*, the sequence contig on which we identified this factor is small (about 2 kbp) and thus the full sequence of this factor is not known.

All 30 ELT-2 orthologues have a single DNA-binding domain. The predicted ELT-2 orthologue of the Clade I nematode *S. baturini* is apparently truncated in its basic domain, with only 13 of 30 typical amino acids, and we were unable to find further coding potential in the DNA that could, for example with a different splicing pattern, account for the missing amino acids. Upstream of the DBDs, a degenerate C4-type zinc finger can be identified in 28/30 ELT-2 orthologues. The size of this domain varies considerably in the number of amino acids between the pairs of cysteines, with as few as 9 amino acids in the case of the Clade V species *Mesorhabditis belari*, to as many as 20 in the Plectid (Clade C) species *P. sambesii*. And unlike the tandem arrangement of DBDs in the ELT-1 orthologues, the divergent upstream zinc finger is found at variable distances, from as few as 34 amino acids upstream in *Trichuris muris*, to as many as 152 amino acids in *Meloidogyne incognita.* The species that lack the degenerate C4 domain are the *C. elegans* relative *C. plicata*, and the Clade I species *R. culicivorax*. Unexpectedly, two predicted ELT-5 orthologues in *P. sambesii* have an upstream degenerate C4 domain.

The ELT-3 and ELT-5 factors have similar one-DBD structures. We found ELT-3 factors in only 22 of 32 species, with orthologues not apparent in *M. incognita* and *P. sambesii*, as well as all Clade I and II species we searched. The ELT-3 orthologs are the shortest GATA factors overall; 14 of the 22 are shorter than 300 amino acids (although two of the sequences are incomplete), and 18/22 end shortly after the basic domain. ELT-5 orthologs were found in 26/32 species, slightly more than with ELT-3. Unlike the ELT-3s orthologues, all of the ELT-5s in the Chromadoria have many additional amino acids after the basic domain, with a range of 38 amino acids in *Steinernema carpocapsae* to as many as 126 in its close relative *Strongyloides ratii.* Only the ELT-5 orthologues in the Clade I species *S. baturini* and *R. culicivorax* end after the basic domain, similarly to most of the ELT-3s.

We also identified regions in the GATA factors shown in Figure 4 that contain segments at least 10 amino acids long in which at least 6 amino acids are serine, a polyserine or Poly-S domain. In prior work on the END-1 and END-3 endoderm-specifying GATA factors in *C. elegans*, we identified highly conserved Poly-S regions in the amino ends of all END-1 and END-3 orthologues, factors that occur only in the Elegans Supergroup [40]. Of the nematode GATA factors, overall, Poly-S regions were apparent in 26/32 ELT-1 orthologues, in a region of about 10–20 amino acids, typically some 25 amino acids upstream of the amino DNA-binding domain. Poly-S domains were also found in the other factors, but less frequently, in only 11/30 ELT-2, 2/22 ELT-3, and 3/26 ELT-5 orthologues. A Poly-S domain was also apparent in *Drosophila Grain*.

As an additional test for the origin of the nematode GATA factors, we searched the genome of the tardigrade *Hypsibius dujardini*, proposed to be a ‘bridge species′ between arthropods and nematodes in the protostomes, and sharing developmental properties similar to both groups [59]. In *H. dujardini*, there are three predicted GATA factors of the two-DBD type, and all three are structurally like vertebrate GATA3 or GATA5 (data not shown).

### 3.4. Introns Reveal Possible Origins of Nematode GATA Factors

Intron positions can be used to infer patterns of GATA factor evolution [25,26,40]. To further aid in comparison of gene structure, the positions of introns in the coding region are indicated by gray triangles directly over the proteins. We have colored a subset of conserved introns that occur in the same positions within the amino basic domains of ELT-1, and near the end of the C4 zinc finger of the DNA-binding domain of ELT-2. (Additional intron positions are also conserved.) As can be seen among the group of ELT-1 proteins, the introns within the two basic domains, shown in purple and blue, are conserved in *Drosophila Grain*, chicken *G. gallus* cGATA1, and the nematode ELT-1 orthologues. The purple-colored intron is found between the 20th and 21st codons after the fourth cysteine of the amino zinc finger, while the blue-colored intron is found between the 8th and 9th codons after the fourth cysteine of the carboxyl zinc finger. The same (blue) intron also occurs in the single zinc finger of 23/26 predicted ELT-5 orthologues.

Whereas the ELT-1 introns are conserved among nematodes, those within the ELT-2 carboxyl zinc finger show differences. Within *C. elegans* ELT-2, an intron occurs 1 bp after the codon for the third cysteine of the (carboxyl) zinc finger. This intron is found in 22/24 ELT-2 factors shown within the Chromadoria, but in none of the species outside of this group, and not in the chicken or *Drosophila* GATA factor genes. This intron also occurs in 19/22 of the ELT-3 factors shown in the figure, but unexpectedly, 5/6 putative ELT-2 orthologues from the more basal Dorylaimia, including the four Trichinellida species and the mermithid *R. culicivorax*, do not have this intron. Instead, they have the intron found in the carboxyl basic domain of the ELT-1 orthologues. These intron patterns lead to a straightforward hypothesis to explain the origin of all four major GATA types (see Discussion).

We note in passing that the GATA factors in *Strongyloides ratti* are unusually devoid of introns. The *S. ratti* ELT-1 retains only the two introns within the basic domains, and the other three shown have only a single intron each. Additional ELT-3 and ELT-5 orthologues from *S. ratti* have no introns in their coding regions (see Supplemental File S1). These results are consistent with the observation that *Strongyloides* genes are known to have experienced a loss of introns across the genome [60].

### 3.5. Extra GATA Factors in D. Coronatus, P. Pacificus and P. Redivivus Are Most Closely Related to ELT-3

The three species *D. coronatus*, *P. pacificus* (Clade V) and *P. redivivus* (Clade IV) each have 10 or more GATA factors. In *D. coronatus*, the extra factors are accounted for by recent paralogous duplicates of ELT-1, ELT-2, and ELT-5, with the extra factors being three paralogues of ELT-3 (Figure 3). In *P. pacificus*, most of the additional GATA factors are similar paralogues of ELT-3, with one of them (ELT-3.2) having two ELT-3-like DBDs spaced 311 amino acids apart; all have the conserved intron in the zinc finger similar to the other ELT-3 orthologues (Figure 5a). In *P. redivivus*, we separated the additional GATA factors into their own category of ELT-X because as a set they have unusual properties (Figure 5b). From Figure 3, their DBDs are similar and appear to have descended from ELT-3. One of them, ELT-X.1, has an unusual ‘CSNSNC′ in the first two cysteines of the C4 zinc finger rather than the typical CXXC. This unusual spacing was previously observed in *C. elegans* END-1 and many of its orthologues but was not found in any of the other nematode GATAs that we identified in this work [40]. Five of the *P. redivivus* ELT-X GATAs have a single DBD, and four have two DBDs spaced by approximately 100 amino acids. In addition, unlike the more ELT-3-like extra GATAs of *P. pacificus*, five of those from *P. redivivus* are intronless, and three have a Poly-S domain. Furthermore, none have the conserved ELT-3 zinc finger intron, and in fact two of them have an intron in the basic domain like the ELT-5 orthologues. The occurrence of so many additional GATA factors has implications for the evolution of gene regulatory networks (see Discussion).

### 3.6. Patterns of Amino Acid Conservation in Nematode GATA Factors

**Figure 6:**
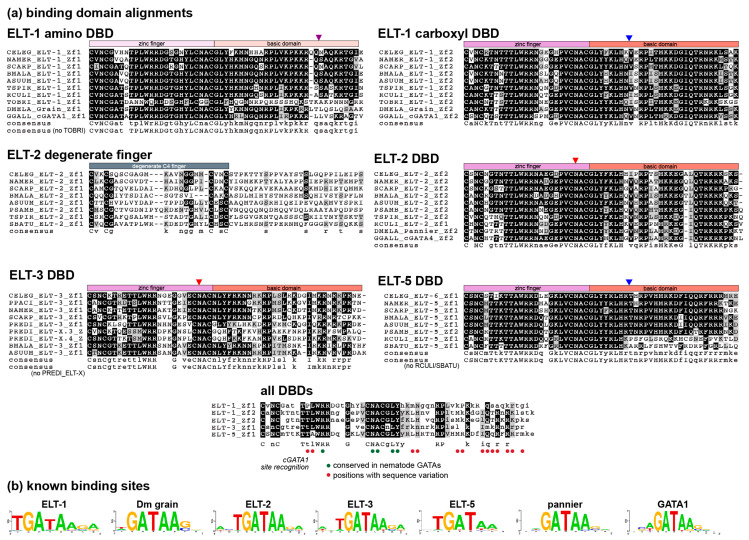
Sequence recognition sites differ among nematode GATA factors. (**a**) Alignment of zinc fingers and adjacent domains of select nematode GATA factors, plus the degenerate C4 zinc finger region of ELT-2. Species were chosen to sample diversity of sequences across the phylum. *Drosophila* and vertebrate GATA factor sequences are included for comparison. Consensus sequences are shown below each alignment block, and an alignment of all the consensus sequences is shown at the bottom. Capital letters indicate absolute conservation, while lowercase indicate majority conservation. Circles are used to indicate the corresponding amino acids known to be involved in direct DNA base recognition in chicken (*G. gallus*) cGATA1 [61]. 13/18 of these positions varied across the nematode GATAs, suggesting that the various factors bind slightly different sequences. White text on a black background indicates identity, while black text on a gray background indicates conservative sequence differences. (**b**) Experimentally determined binding sites for *C. elegans* ELT-1, ELT-2, ELT-3, and ELT-6 (shown as ELT-5), plus examples of *Drosophila* (Dm) and vertebrate GATA factors (human GATA1, nearly identical to cGATA1) from other studies [62,63,64,65,66,67,68]. Binding site diagrams were generated with WebLogo 2.8.2 (https://weblogo.berkeley.edu/). Additional bipartite binding sites are available for ELT-1, Grain, and hGATA1 that contain two GATA sites in inverted orientation; for ease of comparison, we have shown examples of binding motifs that show only a single site.

During the identification of nematode GATA factors it became apparent that there is a high degree of amino acid conservation within their DNA-binding domains. We searched for additional domains that are conserved; however, we did not find any extensive regions of similarity aside from some short serine-rich regions as described below. To demonstrate conservation and divergence within the DBDs, we aligned a subset of these within each class (Figure 6a). As well, although the divergent amino zinc finger region of ELT-2 is not likely to be involved directly in DNA binding, we included these sequences for comparison.

Several generalizations can be made from the alignments. First, each class shows unique patterns of amino acid conservation. Within ELT-1, the first zinc finger almost universally contains the sequence TPLWRR, which was not observed in any of the other GATA classes except for a single-DBD *S. carpocapsae* ELT-3 orthologue. Consistent with the ELT-1 class being homologous with GATA factors outside nematodes, the TPLWRR sequence is also found in the amino DBD of *Drosophila* and vertebrate GATA factors. The highly divergent ELT-1 amino DBD from the basal *Tobrilus* sp. was the only exception to the occurrence of TPLWRR; however, this orthologue shows lack of conservation throughout the amino DBD. If we exclude *Tobrilus* and consider only the nematode GATA factors shown, there is conservation at 38/55 (69%) positions in the zinc finger and associated basic domain. The carboxyl DBD of ELT-1 is also highly conserved and contained a TTLWRR sequence shared with the *Drosophila* and vertebrate carboxyl zinc fingers. Over the carboxyl DBD, the nematode ELT-1s shown have 36/55 (66%) amino acids conserved. 

Among the ELT-2 orthologues, the amino C4 finger is poorly conserved outside of the four cysteines, consistent with this domain being retained for some function that does not involve direct recognition of nitrogenous bases in DNA. The carboxyl C4 finger, in contrast, is highly conserved, containing a characteristic TTLWRR found in the carboxyl finger of the ELT-1 orthologues, and shared with vertebrate DBDs that recognize the GATA motif. Here the conservation can be seen to be a bit weaker within the adjacent basic domain. Overall, the carboxyl DBD of the nematode ELT-2 DBDs shown have 33/55 (60%) conserved positions, slightly less than with the carboxyl ELT-1 DBDs.

Among the single-finger GATAs, conservation is much higher for ELT-5 than ELT-3. The ELT-5 orthologues show conservation of a diagnostic TTAWRR sequence and high conservation overall except in the basic domains of the more basal Clade I nematodes (*R. culicivorax* and *S. baturini*) which show low conservation even with each other. Excluding these species, the ELT-5 DBDs show 42/55 (76%) conserved positions, but this is reduced to 25/55 (45%) if the Clade I orthologues are included. In contrast, both the zinc finger and basic domains of the ELT-3 orthologues showed the lowest conservation among nematode GATAs. In the alignment in Figure 6 we included two of the *P. redivivus* divergent ‘ELT-X’ factors, to show that they share sequence similarity with the ELT-3 orthologues, consistent with their placement in the phylogeny shown in Figure 3. Excluding these ELT-X factors, conservation across the ELT-3 DBDs shown was only 19/55 (35%). Hence, these GATAs appear to be where divergence is occurring more frequently.

Overall, an alignment of the consensus sequences from the figure shows that identical amino acids occur at only 15/55 (27%) of positions. From the solution structure of cGATA1, we have indicated the amino acid positions that are likely to directly mediate base pair recognition, and hence transcription factor specificity [61]. Of these, 13/18 (72%) vary among the nematode GATAs, consistent with structural divergence potentially influencing the optimal binding site sequences among the nematode GATA factors. Finally, in Figure 6b, experimentally identified binding sites for the *C. elegans* orthologues of the nematode GATA factors and representative *Drosophila* and vertebrate factors are shown to demonstrate that, while the core ‘GATA′ motif recognized by these factors remains conserved, there is experimentally known variation in the optimal binding sites.

### 3.7. Possible Conserved Regulatory Regions in Flanking Regions

In a prior work, we identified conserved *cis-*regulatory sites in the upstream flanking regions of the MED and END genes within the Elegans Supergroup [40]. In that case, the gene network under study is a derived blastomere specification network for which prior regulatory sites had already been established in *C. elegans*, and which were demonstrated to be functionally conserved in closely related species [36,69]. To look for possible *cis-*regulatory motifs at the level of the Nematode phylum, we used the MEME algorithm [54] to identify overrepresented sequences that could represent binding sites for regulatory factors within the upstream 1000 bp of 5′ flanking sequences within each GATA class. Eight motifs were found in the regulatory regions of the genes encoding the ELT-1, ELT-2, and ELT-5 factors (Figure S3). These include an unusual ‘double-GATA′ motif identified in 10 species for ELT-2, which consists of two HGATAR sites in an inverted, convergent orientation. Within *C. elegans*, a pair of tandem (direct) GATA sites mediates positive autoregulation of ELT-2 [70]. Hence, it is possible that this double-GATA site may have a similar function in mediating autoregulation in some species. Besides this motif a polypyrimidine sequence found in some promoters of genes encoding ELT-1 (17/29 species) or ELT-5 (17/23 species). A similar polypyrimidine sequence was observed in some Elegans Supergroup genes encoding the END-1 and END-3 factors [40]. This sequence, and others that were found, remain unknown significance.

We also examined the putative 3′ flanking regions for possible motifs that might represent sites of post-transcriptional regulation when present in the 3′ untranslated region of mRNAs. Nine conserved motifs were found, including polypyrimidine tracts in ELT-1 and ELT-5 orthologues (Figure S4), suggesting these could be important for gene regulation. However, it is not clear what role these motifs may play, especially for the pyrimidine tracts which were found in both promoters and putative 3′UTRs.

## 4. Discussion

### 4.1. Evolution of the GATA Factors in Nematodes

**Figure 7:**
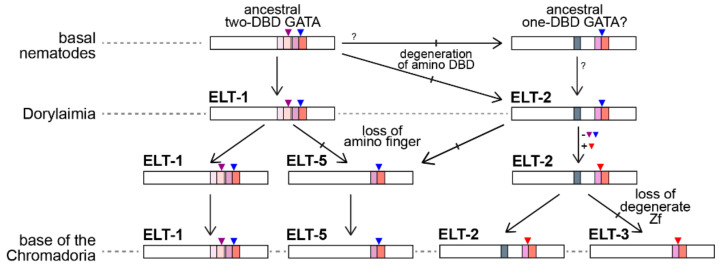
Model for GATA factor evolution in nematodes. An ancestral two-DBD vertebrate-like GATA factor was present at the base of the phylum. An ELT-2-like factor may have arisen in early nematodes or else was inherited from a nematode-arthropod common ancestor. The present-day Dorylaimia retain versions of these ancestral ELT-1 and ELT-2 factors. An ELT-5 progenitor arose from ELT-1 or ELT-2 through duplication followed by the loss of the amino finger. In parallel, the ELT-2 progenitor underwent a change in its introns within the DNA-binding domain. A duplicate of ELT-2 that lost the degenerate C4 domain became a progenitor for present-day ELT-3. The Chromadoria retain orthologues of ELT-1, ELT-2, ELT-3, and ELT-5. (Symbols are colors are as in Figure 4).

In this work we have presented a phylum-wide overview of the GATA factor family of transcription factors in nematodes and we hereby propose a model to explain their evolution (Figure 7). From the structural similarity of nematode GATAs with those of *Drosophila* and vertebrates, there may have been only one conserved GATA factor at the base of the nematodes that retains highest conservation with present-day ELT-1 orthologues. Among basal nematodes, or in an ancestor of nematodes with arthropods, an ELT-1 factor was a likely progenitor of an ancestral ELT-2-like gene. Evidence for this model is that Trichinellids, a subset of the Dorylaimia, retain an ancestral intron in their DBD that is retained in present-day ELT-1 and both *Drosophila* and vertebrate GATAs. However, whereas the amino finger of ELT-1 has a GATA DBD structure, the ELT-2s of most species retain a poorly conserved degenerate amino zinc finger that presumably has lost its DNA-binding function. Outside of the Trichinellids, we found evidence for ELT-2 and ELT-5-like factors in *S. baturini* and *R. culicivorax*. The Plectid *P. sambesii*, at the base of the Chromadoria, also appears to retain ELT-1, ELT-2, and ELT-5-like factors. The presence of the ELT-1-type intron in the ELT-5 orthologues is consistent with ELT-5 having been derived from an ELT-1 or early ELT-2-like progenitor by loss of the upstream finger. The *P. sambesii* ELT-5-like protein has a small degenerate upstream C4 zinc finger that might be consistent with evolution of ELT-5 from an ELT-2 duplicate, as opposed to ELT-1.

At the base of the Chromadoria, among the Spirurina (Clade III), Tylenchina (Clade IV), and the Rhabditina (Clade V), we were able to almost always identify orthologues for all four of ELT-1, ELT-2, ELT-3, and ELT-5. At some point at the base of the Clade III-IV-V group, the genes encoding the ELT-2 orthologues lost the intron found in the carboxyl DBD of ELT-1 and gained a different intron just after the third cysteine in the zinc finger. This intron is present among nearly all of the ELT-2 and ELT-3 factors in the Chromadoria, consistent with ELT-3 having arisen from duplication of an ancestral ELT-2 at the base of this group followed by complete loss of the degenerate C4 finger. 

The occurrence of many more GATA factors, particularly in Clades IV and V, is consistent with further duplication and divergence. There is a precedent in frequent duplication of the derived END-3 and MED factors that occurred among species in the Elegans Supergroup [40]. Indeed, the single-DBD nature of these factors suggests that it is the single-finger ELT-3-like factors that are most likely to undergo further duplication. This was observed in *P. pacificus*, which has 10 GATA factors overall, and among the unusually expanded 14 factors in *P. redivivus*. In the latter case, many of the ELT-3-like factors were sufficiently diverged from ELT-3 so that we grouped them together as ‘ELT-X′ factors.

A search for putative sequence motifs that could mediate transcriptional or post-transcriptional regulation revealed some putative sites, most notably an inverted repeat GATA binding site that could mediate autoregulation of the ELT-2 factor genes. However, we were otherwise unable to associate identified sequences with possible regulators, and it is not clear that some of the sequences (e.g., a ‘polypyrimidine’ motif found among the ELT-1 and ELT-5 factor promoters and also 3′UTRs) represent *bona fide* sites that mediate regulation. It may be that sequence motifs representing sites of GATA factor regulation at the transcriptional or post-transcriptional levels are either not deeply conserved across the phylum, or they are below the identification threshold for detection by a sequence-based method alone.

### 4.2. What Do the Nematode GATA Factors Do?

The collective set of functions for the *C. elegans* GATA factors suggest that just as in other animals, the nematode GATA factors function in specification of progenitor cells and expression of target genes involved in tissue-specific functions. However, many of these are derived, particularly in endoderm specification [40]. Instead, we can look at the ‘core’ ELT-1, ELT-2, ELT-3, and ELT-5 factors conserved with other nematodes to deduce how their functions might be conserved.

First, ELT-1 is essential for specifying epidermal progenitor cells [33]. ELT-3 has a similar expression pattern to ELT-1 and can also specify epidermal fates in the absence of ELT-1 [31]. ELT-1 also has a role in sperm function [71]. In executing the hypodermal fate, ELT-1 and ELT-3 likely bind to hundreds of target genes [72]. Hence, we propose that the ELT-1 factors likely have similar functions among other nematodes in activation of genes in the epidermis. The additional role of *C. elegans* ELT-1 in sperm function is intriguing, and it will be of interest to see if sperm expression of ELT-1 is common across other nematodes. Because ELT-3 and ELT-1 may have similar functions in *C. elegans*, the nematode ELT-3 orthologues may also be conserved in having a role in epidermal cell fates. However, because they share the least amount of DBD sequence conservation (Figure 6a), the role of ELT-3 as an ELT-1 reinforcer may be divergent (see below).

In *C. elegans*, ELT-2 is essential for the differentiation of the intestine, and when expressed in naïve cells can specify the intestinal fate [32]. Most of the Elegans Supergroup species contain an additional derived GATA factor, ELT-7, that enforces some of ELT-2 functions [38,40]. A conserved role for ELT-2 in intestinal differentiation comes from analysis of an apparent ELT-2 orthologue in the Clade V species *H. contortus*, which can promote intestinal development when forcibly expressed in *C. elegans* [73]. An intestine-enriched ELT-2 orthologue from the Clade III species *Ascaris suum* has also been identified [74]. ELT-2 is important for other aspects of intestine function, including longevity, digestion, stress response, and immunity in *C. elegans* [75,76,77,78,79]. Given the myriad of target genes that must be expressed in response to ELT-2, and the observation of ELT-2-like genes in *H. contortus* and *A. suum*, it is most likely that ELT-2 has retained an essential function in executing intestinal cell fates across nematodes.

A logical comparison for ELT-2 is the *Drosophila Serpent* protein, which, like *C. elegans* ELT-2, is involved in the development of the gut, and also in hematopoiesis [19,30]. The *C. elegans* gut is a simple tube of a small number of cells that is maintained for the life span [80,81]. In contrast, the intestine of *Drosophila* is completely replaced in larval development and maintains a pool of stem cells [82]. The longest *Serpent* isoform has two tandem GATA zinc fingers that more closely resemble those of ELT-1. Indeed, the carboxyl zinc finger of *Serpent* has the sequence TSLWRR, which occurs in some putative ELT-3 orthologues in nematodes. As noted above, however, all nematode ELT-2 orthologues have TTLWRR. Hence, the role of ELT-2 and *Serpent* in intestine development may be convergent.

Finally, ELT-5 in *C. elegans* exists as two paralogues with overlapping function in specifying cell fates in epidermis, EGL-18 and ELT-6 [83,84]. Loss of *egl-18* and *elt-6* together results in a larval lethal phenotype. Given the high degree of conservation in the DNA-binding domains of these factors, we hypothesize that ELT-5-like GATA factors likely have a role in executing some aspect of postembryonic cell fates.

### 4.3. GATA Factor Expansion Favors ELT-3-Like Factors

GATA factor expansion appears to be rare among nematode species, which generally maintain fewer than six such factors in total (Figure 1). A subset of the species examined in Clades IV-V have at least ten GATA factors, namely *D. coronatus*, *P. pacificus*, and *P. redivivus* (Figure 1 and Figure 5). In all but *P. redivivus*, extra factors tended to be ELT-3-like, having the conserved intron found in the *C. elegans* ELT-3 and ELT-2 zinc fingers. In *P. redivivus*, a subclass of ELT-3-like factors exhibiting diverse structure and sequences were collectively grouped as ELT-X (Figure 5b). If we consider that END-1, END-3, and ELT-7, as derived GATA factors in the Elegans Supergroup, resemble ELT-3 in having the same zinc finger intron in a single DNA-binding domain, with little sequence afterwards, the expanded GATA factors in *C. elegans* are also ELT-3-like. We hypothesize, therefore, that when GATA factor duplication and divergence occur, they are more likely to occur with ELT-3-like GATA factors. There may be something about the ELT-3-like DNA-binding domain that makes it more flexible to evolutionary change. A precedent for this is the expansion of the endoderm specification network in the Elegans Supergroup to form a cascade of factors that operate in a feed-forward gene network that ends in ELT-2 [40]. It may be, therefore, that the *P. redivivus* expanded ELT-X factors may function in a derived regulatory network for some developmental process.

## 5. Conclusions

We conclude that, among nematodes, there has been maintenance of at least one two-fingered GATA factor from a common ancestor shared with arthropods and vertebrates. Further expansion by duplication and divergence resulted in a conserved set of four GATA factors among most species in the Chromadoria. Additional expansion of GATA factors is rare and results preferentially from duplication and likely divergence of single-fingered factors like ELT-3. The high degree of sequence conservation, particularly among the ELT-1, ELT-2, and ELT-5 factors, suggests similar roles of these factors in cell specification and execution of differentiation programs and tissue-specific functions. Future work could shed light on the possible conserved function, particularly tissue and stage gene expression analysis across the phylum. Additional genome sequencing of species in the more basal clades could also resolve whether the ELT-2 factors, with their unusual upstream degenerate C4 zinc finger, were present at the base of nematodes or arose later from duplication of an ELT-1-like progenitor. 

## Supplementary Materials

The following are available online at https://www.mdpi.com/2221-3759/8/4/27/s1, Figure S1: Linkage among a subset of genes encoding paralogous GATA factors within species. Sequence scaffold names are shown and the relative distances between the start of each gene are approximately to scale, Figure S2: RAxML-NG tree that includes the *Tobrilus* sp. ELT-1-like GATA factor and the partial *Bathylaimus* sp. GATA sequence., Figure S3: Putative upstream regulatory motifs identified by MEME in multiple species with an E-value smaller than 0.05, Figure S4: Putative post-transcriptional regulatory motifs in the 1000 bp immediately downstream of the predicted stop codon identified by MEME and with an E-value smaller than 0.05, Table S1: Predicted GATA factors from 32 nematode species in multiple species, and corresponding flanking nucleotide sequences, File S1: Sequences of putative GATA factors and flanking DNA.
