# Supplementary material for: Evolution of Developmental GATA Factors in Nematodes
Source: J Dev Biol. 2020 Nov 16;8(4):27. doi: 10.3390/jdb8040027 (PMC7712238; doi:10.3390/jdb8040027)
Supplement: Supplementary file 1 [file jdb-08-00027-s001.zip › jdb-987434 - supplementary/jdb-987434 - supplementary figures.docx]

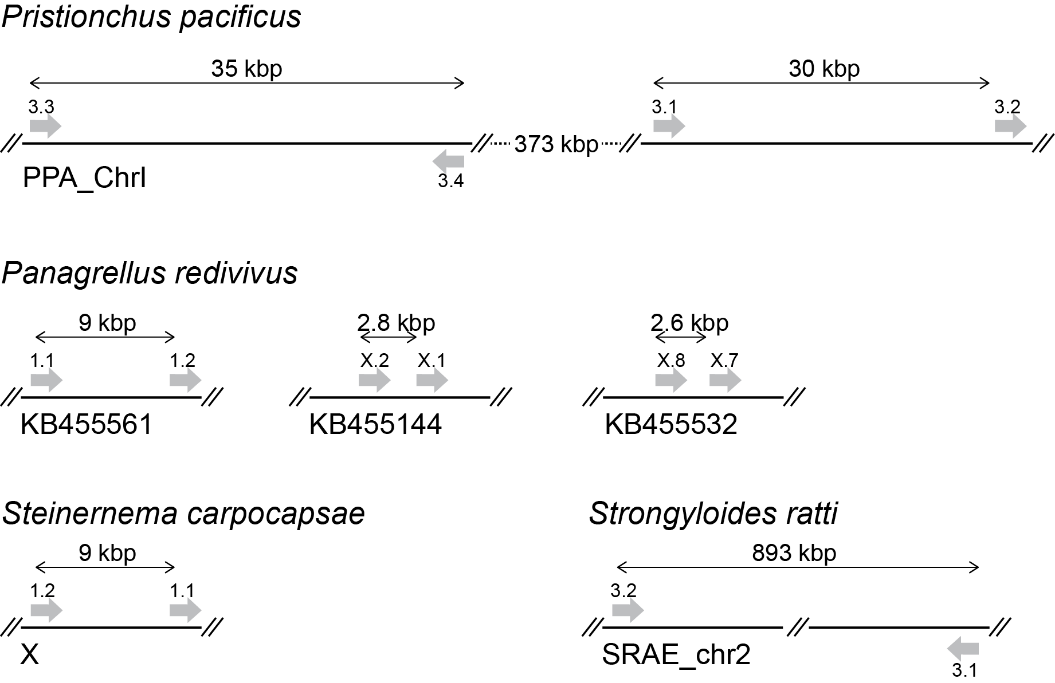


**Figure S1.** Linkage among a subset of genes encoding paralogous GATA factors within species. Sequence scaffold names are shown and the relative distances between the start of each gene are approximately to scale.


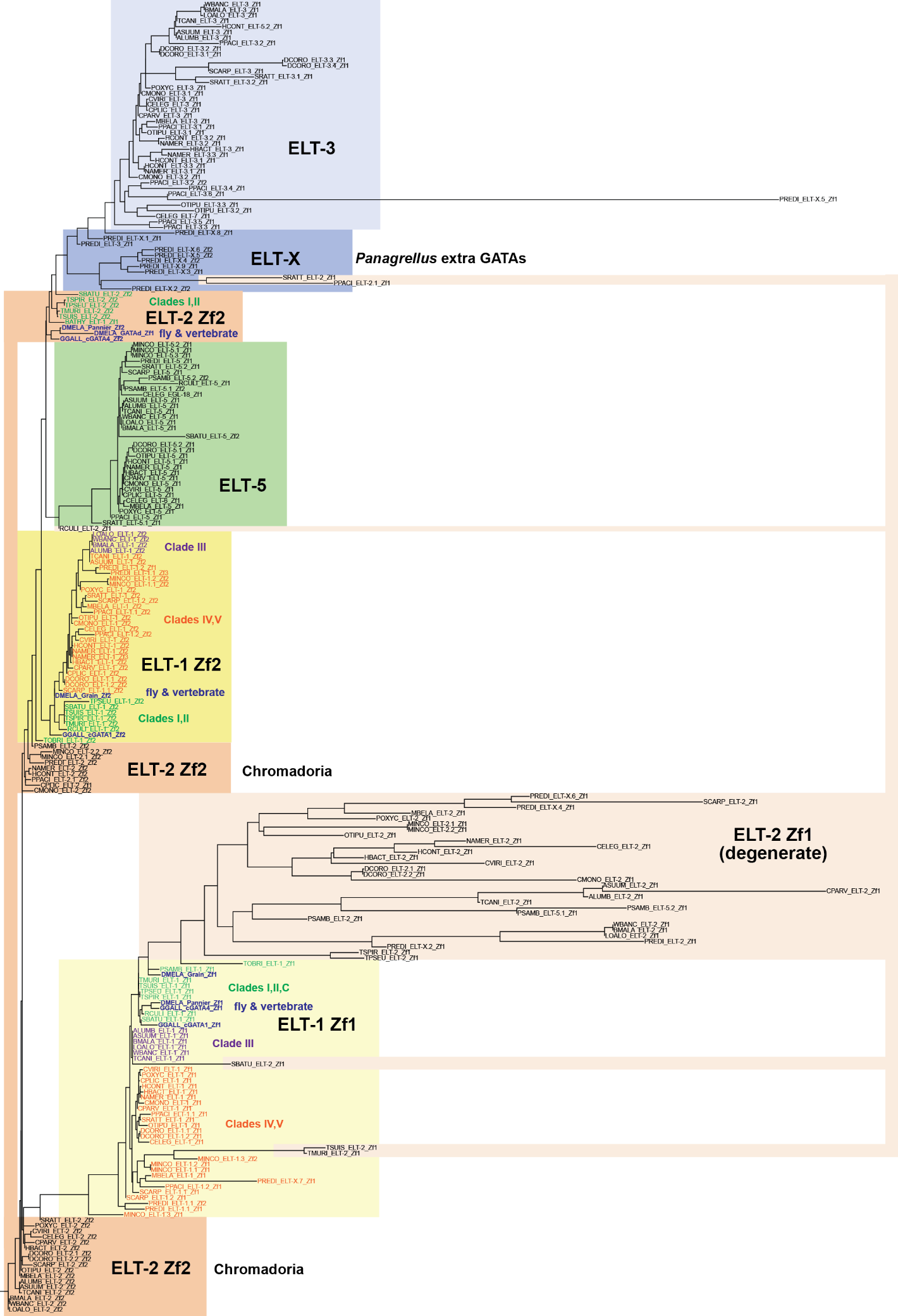


**Figure S2.** RAxML-NG tree that includes the *Tobrilus sp.* ELT-1-like GATA factor and the partial *Bathylaimus sp.* GATA sequence.


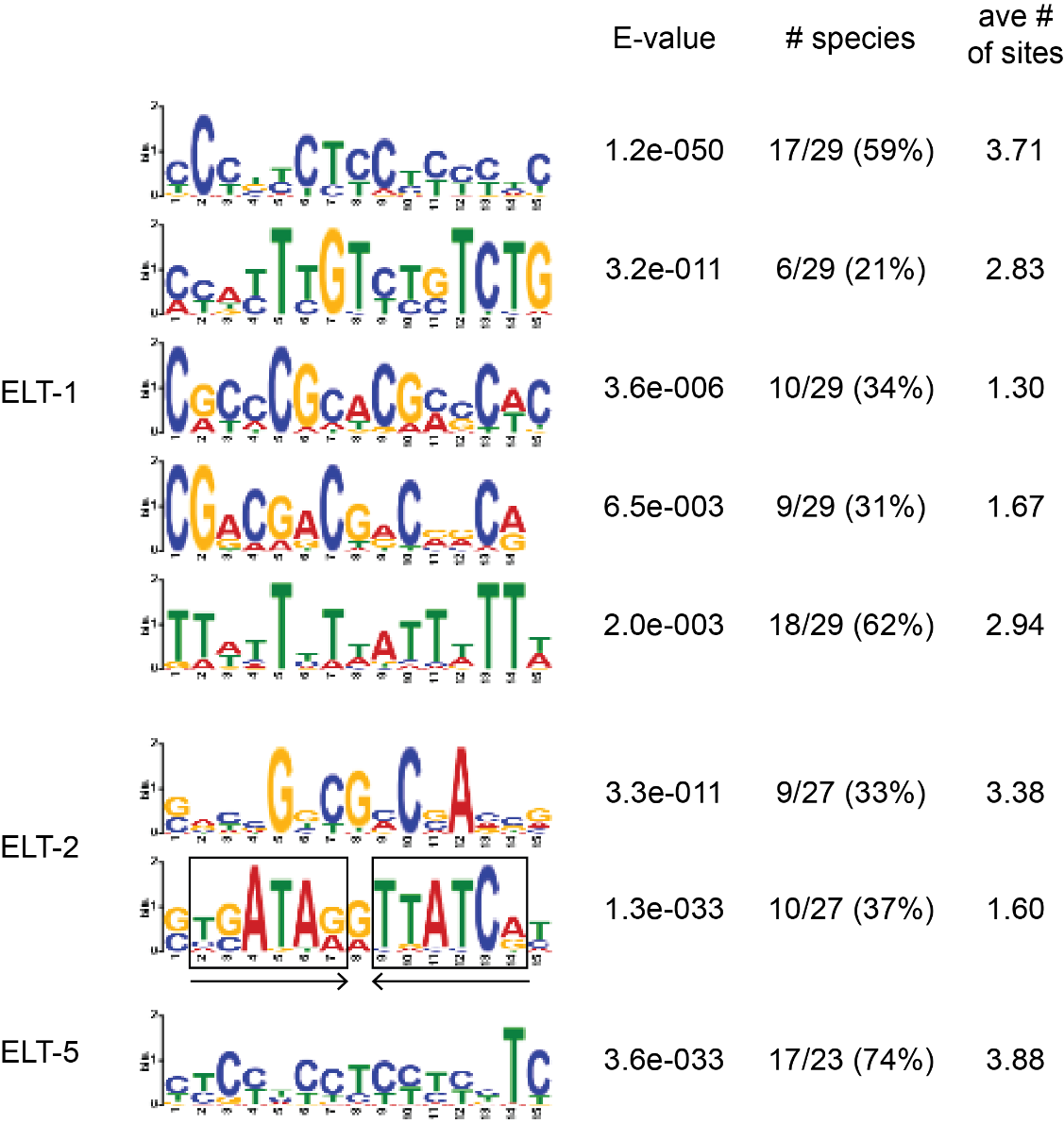


**Figure S3.** Putative upstream regulatory motifs identified by MEME in multiple species with an E-value smaller than 0.05. For the second ELT-2 region, putative inverted HGATAR regions are indicated by rectangles on the sequence motif. No motifs with an E-value < 0.05 were found for ELT-3. Among the ELT-2 promoters, we omitted *A. lumbricoides* because it had < 10 bp of sequence in its scaffold, and *M. belari* and *M. incognita*, because these each contained unique, highly repetitive regions that were not found in the corresponding orthologues of other species.


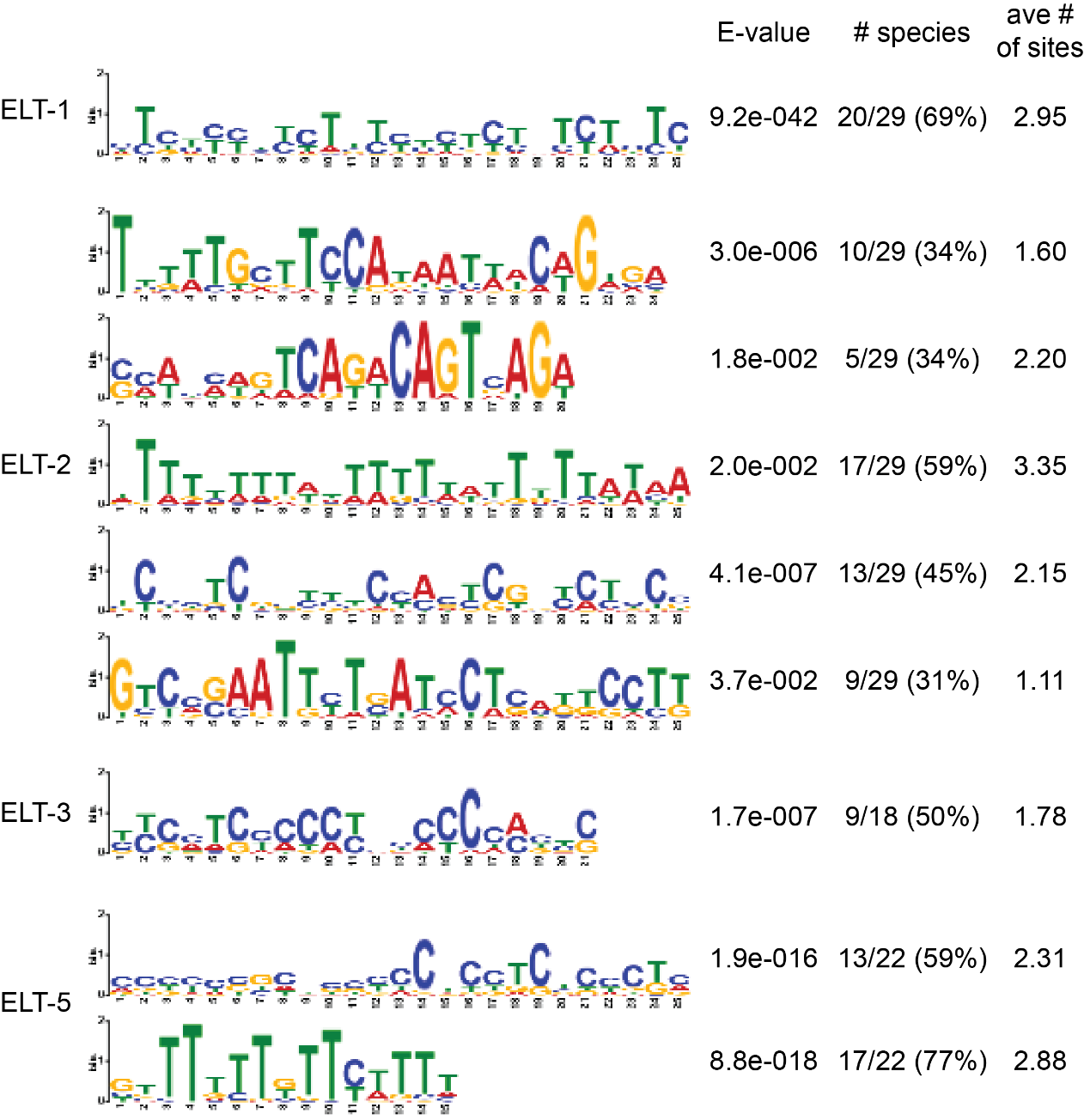


**Figure S4.** Putative post-transcriptional regulatory motifs in the 1000 bp immediately downstream of the predicted stop codon identified by MEME and with an E-value smaller than 0.05. We omitted the 3'UTRs of *M. belari* ELT-2, *T. canis* ELT-3, and *C. elegans* ELT-6, as these each contained unique, highly repetitive regions that were not found in the corresponding orthologues of other species.

**Table 1.** Predicted GATA factors from 32 nematode species in multiple species, and corresponding flanking nucleotide sequences.
